# Clinical and histopathologic characteristics and outcomes of cellular dermatofibroma: A retrospective observational study of 29 patients

**DOI:** 10.1016/j.jdin.2024.12.001

**Published:** 2025-01-06

**Authors:** Nicholas T. Le, Daniel B. Eisen

**Affiliations:** a University of California Davis School of Medicine, Sacramento, California; b Department of Dermatology, University of California Davis, Sacramento, California

**Keywords:** cellular dermatofibroma, cellular fibrous histiocytoma, dermatofibroma, excision, metastasis, recurrence

*To the Editor:* Cellular dermatofibromas (CDF) constitute an infrequent type of dermatofibroma that tend to recur and rarely metastasize. Currently, there is no clear consensus on the best treatment approach for these lesions. Recommendations have ranged from observation to wide local excision or Mohs micrographic surgery, but uniform consensus has not been achieved.^1^ Given the lack of clarity and concrete guidelines surrounding diagnosis and treatment, we aim to describe the procedures performed, identify clinical and histopathologic characteristics, outcomes after diagnosis, recurrence rate, metastatic potential, and mortality, if any, of CDF.

An Institutional Review Board-approved retrospective analysis of medical records and skin biopsies of 3885 patients with dermatofibromas in the University of California Davis Health system between April 2009 to April 2024 was conducted. Through manual screening, 29 of 3885 patients had a confirmed diagnosis of CDF through pathology. Baseline demographics, clinical and histopathologic features, differential diagnosis, treatments, and outcomes were collected from patient’s charts and pathology reports.

Among the 29 CDF patients, 79.3% were female and the mean age was 47.1 (standard deviation (SD): 16.1) (Table I). Immunohistochemical staining was done in 15 patients (Table II). Nineteen of 29 (65.5%) patients were initially treated by dermatology, while 10 of 29 (34.5%) patients were initially treated by primary care. Initial management strategy included 1 patient treated by shave removal, 22 treated by biopsy, and 6 treated by excision. 26 of 29 cases reported initial lesion size of CDF (Average: 11.0 mm, Range: 31.0 mm, SD: 6.3 mm). Twenty-one patients had CDF removed through biopsy or treatment after biopsy. Eight did not have it removed including one patient lost to follow-up. Treatment modalities after biopsy included 10 excisions, 2 punch excisions, and 17 observations. Time from diagnosis at biopsy to treatment was on average 79.9 days with a range of 298 and SD of 80.5 days. Seven of 12 excision cases provided surgical margins. Surgical margins for punch excisions were not reported. Average surgical margins for excision were 3.4 mm with a range of 3 mm and SD of 1.4 mm. Recurrence was defined as reappearance of a lesion after it was removed. 3 of 21 (14.3%) treated patients had local recurrence, all on upper extremities. 2 of 3 recurrences reported sizes (average: 6.8 mm, Range: 2.5 mm, SD: 1.8 mm) and received treatments of observation and excision, respectively. Average days till recurrence were 750.7 days with a range of 1347 and SD of 719.3 days. One of 3 recurrent patients had positive margins on original biopsy; the other 2 cases did not report margins. Zero of 29 patients had metastasis or mortality.

**Table I:** Patient demographics and CDF characteristics

Age (y), mean ± standard deviation	47.1 ± 16.1 (Range: 21-83)
Gender	
Female	23 (79.3%)
Male	6 (20.7%)
Ethnicity	
Non-Hispanic	21 (72.4%)
Hispanic	4 (13.8%)
Unknown/not reported	4 (13.8%)
Race	
White	18 (62.1%)
Asian	3 (10.3%)
Native Hawaiian or other Pacific Islander	1 (3.5%)
Black or African American	1 (3.5%)
Unknown/not reported	6 (20.7%)
Location of cellular dermatofibroma	
Upper extremities	7 (24.1%)
Lower extremities	12 (41.4%)
Cheek	3 (10.3%)
Back	2 (6.9%)
Scalp	1 (3.5%)
Neck	1 (3.5%)
Axilla	1 (3.5%)
Auricular	2 (6.9%)
Favored diagnosis∗	
Dermatofibroma	13 (44.8%)
Neoplasm of uncertain behavior of the skin	13 (44.8%)
Subcutaneous nodule	1 (3.5%)
Squamous cell carcinoma	1 (3.5%)
Cyst	1 (3.5%)
Outcomes	
Local recurrence	3 (10.3%); local recurrence occurred after excisional biopsy, excision, and shave removal, respectively
Regional recurrence	0 (0%)
Metastasis	0 (0%)
Death	0 (0%)

All percentages in Table I were calculated based off 29 patients with CDF.

*CDF*, Cellular dermatofibromas.

∗ Favored Diagnosis is referring to a clinical differential diagnosis.

**Table II:** Immunohistochemical markers of biopsied CDF

Immunohistochemical marker	Positive	Negative	Total studied
CD34	4 (all weak focal positive)	10	14
Factor 13A	8 (1 scattered weak positive; 1 patchy positive; 1 variably positive)	1	9
Desmin	0	1	1
S100	0	7	7
SMA	3 (1 multifocal positive)	0	3
Keratin (MNF116)	0	1	1
MITF	0	1	1
CD68	1	0	0
CD10	1	0	0
CD31	0	2	2
SOX-10	0	3	3
AE1/AE3 (pancytokeratin)	0	3	3
EMA	0	1	1

*CDF*, Cellular dermatofibromas; *EMA*, epithelial membrane antigen; *MITF*, micropthalmia associated transcription factor; *SMA*, smooth muscle actin; *SOX-10*, Sry-related HMg-Box gene 10.

The findings of this retrospective observational study emphasize the distinct characteristics, clinical progression, treatment strategies, and outcomes of CDF, which are underrepresented in literature due to its rarity. This study is limited by its small sample size and single-institutional database. From our findings, recurrence is not unusual, but significant adverse outcomes appear to be absent. While further studies are warranted to examine whether these findings are consistent with other populations, this study provides essential data on clinical outcomes of CDF.

## Conflicts of interest

None disclosed.
